# The correlation between serum endothelin-1 levels and aortic stiffness in patients with non-dialysis chronic kidney disease

**DOI:** 10.1080/0886022X.2026.2641961

**Published:** 2026-03-12

**Authors:** Ssu-Chin Lin, I-Min Su, Chung-Jen Lee, Yu-Hsien Lai, Bang-Gee Hsu

**Affiliations:** a Institute of Medical Sciences, Tzu Chi University, Hualien, Taiwan; b Division of Nursing, Department of Medicine, Hualien Armed Forces General Hospital, National Defense Medical Center, Hualien, Taiwan; c Department of Anesthesiology, Dalin Tzu Chi Hospital, Buddhist Tzu Chi Medical Foundation, Chiayi, Taiwan; d School of Medicine, Tzu Chi University, Hualien, Taiwan; e Department of Nursing, Tzu Chi University, Hualien, Taiwan; f Division of Nephrology, Hualien Tzu Chi Hospital, Buddhist Tzu Chi Medical Foundation, Hualien, Taiwan; g Department of Pharmacology, School of Medicine, Tzu Chi University, Hualien, Taiwan

**Keywords:** Endothelin-1, carotid–femoral pulse wave velocity, aortic stiffness, chronic kidney disease

## Abstract

Increased aortic stiffness (AS) is a recognized predictor of cardiovascular morbidity and mortality in patients with chronic kidney disease (CKD), and endothelin-1 (ET-1), a potent vasoconstrictor, has been associated with vascular dysfunction and atherosclerosis. This study examined the relationship between serum ET-1 levels and AS in patients with non-dialysis CKD. A total of 232 adults with CKD stages 3–5 were enrolled. Fasting blood samples were obtained to measure serum ET-1 concentrations using an enzyme immunoassay, and AS was assessed by carotid–femoral pulse wave velocity (cfPWV) measured using cuff-based volumetric displacement, with cfPWV >10 m/s defining AS. Among the participants, 75 patients (32.3%) were classified as having AS. Compared with those without AS, patients with AS were older and had higher prevalences of diabetes mellitus and hypertension, as well as significantly higher serum ET-1 levels, blood pressure, fasting glucose, HbA1c, and urine protein-to-creatinine ratio. Serum ET-1 levels were positively correlated with cfPWV and remained independently associated with cfPWV in multivariable linear regression analysis. Furthermore, multivariable logistic regression identified serum ET-1, age, diastolic blood pressure, and diabetes mellitus as independent factors associated with AS, with consistent results observed in bootstrap analyses. These findings demonstrate an independent association between serum ET-1 levels and increased cfPWV and AS in patients with non-dialysis CKD.

## Introduction

Chronic kidney disease (CKD) is an increasingly common global health problem and a major contributor to poor clinical outcomes [1]. As renal function declines, the risk of cardiovascular disease rises sharply, with aortic stiffness (AS) playing a key pathological role. In CKD, AS results from atherosclerotic plaque formation and arteriosclerotic changes, including medial calcification and structural remodeling [2]. Increased AS and carotid intima–media thickening are therefore considered important markers of vascular injury in this population [3]. Although contemporary medical treatments target atherosclerotic processes and vascular calcification, patients with CKD continue to experience a disproportionately high burden of cardiovascular risk [4].

Arterial stiffening arises from combined functional and structural changes within the vessel wall, leading to diminished vascular compliance and reduced capacity to dampen pulsatile hemodynamic stress. Even though this process is partly driven by aging, cardiometabolic factors, such as hypertension, diabetes mellitus, oxidative stress, and chronic inflammation, can markedly accelerate its progression [5]. As AS increases, large arteries lose compliance, allowing greater transmission of pulsatile pressure to the microvasculature and promoting cardiovascular injury [6]. Carotid–femoral pulse wave velocity (cfPWV) is recognized as the noninvasive gold-standard measure for AS. Techniques such as applanation tonometry enable the assessment of cfPWV in clinical settings, although they do not directly visualize the aorta [7]. Longitudinal studies in patients with CKD without overt cardiovascular disease have shown that each 1 m/s increase in cfPWV significantly increases the risk of major cardiovascular events during follow-up [8], supporting AS as an early and sensitive cardiovascular risk indicator in CKD before symptoms appear.

Endothelin-1 (ET-1) is an endothelium-derived peptide and one of the most potent endogenous vasoconstrictors [9]. It regulates vascular tone and remodeling by activating ETA and ETB receptors [10]. ET-1 production increases in the presence of cardiovascular risk factors, including hypertension, diabetes mellitus, dyslipidemia, inflammation, and oxidative stress [11]. Consequently, elevated circulating ET-1 levels are consistently observed in vascular disorders such as hypertension, atherosclerosis, heart failure, and acute coronary syndromes, reflecting endothelial dysfunction [12]. In CKD, reduced renal clearance and chronic endothelial activation further increase ET-1 expression [13]. ET-1 contributes to arterial stiffening by promoting vasoconstriction, vascular smooth muscle cell proliferation, and extracellular matrix accumulation [14,15], making it both a biomarker and a potential pathogenic driver of AS in CKD.

While ET-1 is known to contribute to endothelial dysfunction and vascular remodeling, its clinical significance in patients with non-dialysis CKD remains incompletely characterized. Few studies have examined whether elevated ET-1 is associated with early vascular changes prior to dialysis initiation. This study, therefore, evaluated the association between serum ET-1 levels and AS in adults with non-dialysis CKD. We hypothesized that higher ET-1 concentrations would be associated with greater AS. If confirmed, serum ET-1 may serve as a useful marker for identifying CKD patients at elevated cardiovascular risk prior to advanced disease progression.

## Materials and methods

### Participants

This study enrolled 232 adults (aged >18 years) with stage 3–5 CKD from the nephrology outpatient clinic of a medical center in Hualien, Taiwan, from February 2022 to December 2022. CKD was confirmed using two estimated glomerular filtration rate (eGFR) measurements taken at least 3 months apart, calculated using the Chronic Kidney Disease Epidemiology Collaboration (CKD-EPI) equation. CKD severity was categorized according to Kidney Disease Outcomes Quality Initiative definitions, with stage 3 defined by an estimated glomerular filtration rate (eGFR) of 30–60 mL/min/1.73 m^2^, stage 4 by 15–29 mL/min/1.73 m^2^, and stage 5 by values below 15 mL/min/1.73 m^2^ [16]. All participants were managed according to standard multidisciplinary CKD care programs, which included dietary counseling on sodium and protein restriction and guidance to avoid nephrotoxic exposures.

Exclusion criteria comprised active malignancy, chronic inflammatory disease, acute decompensated heart failure, acute coronary events, chronic obstructive pulmonary disease, dependence on maintenance dialysis, or planned kidney transplantation within the subsequent 6 months. Participants who declined informed consent were not enrolled. Diabetes mellitus was identified by fasting plasma glucose ≥126 mg/dL or current antidiabetic therapy [17], while hypertension was defined as blood pressure ≥140/90 mmHg or the use of antihypertensive agents [18].

This study was conducted in accordance with the principles of the Declaration of Helsinki. The Institutional Review Board of Hualien Tzu Chi Hospital, Buddhist Tzu Chi Medical Foundation, approved the study protocol (IRB108-219-A). Written informed consent was obtained from all participants before enrollment.

### Anthropometric measurements

Body weight and height were measured using standardized equipment while participants wore light clothing and no footwear. Body mass index was calculated as body weight (kg) divided by the square of height (m^2^).

### Biochemical measurements

Fasting venous blood samples (≥8 h) were collected from all participants. Part of each sample was used for complete blood count analysis (Sysmex XS-1000i, Sysmex America, Mundelein, IL, USA). The remaining serum was centrifuged at 3000 × g for 10 min and stored at 4 °C within 1 h before analysis. Routine biochemical parameters, including blood urea nitrogen (BUN), creatinine, fasting glucose, glycated hemoglobin (HbA1c), albumin, total cholesterol, triglycerides, low-density lipoprotein cholesterol, total calcium, and phosphorus, were analyzed using an automated chemistry analyzer (Advia 1800; Siemens Healthcare GmbH, Henkestr, Germany). A random urine sample was collected to calculate the urine protein-to-creatinine ratio (UPCR). Serum ET-1 concentrations were measured using a commercially available enzyme immunoassay kit (Biomedica Immunoassays, Biomedica Medizinprodukte GmbH, Vienna, Austria), according to the manufacturer’s instructions.

### Measurement of blood pressure and cfPWV

After a brief seated rest period, brachial blood pressure was measured with a cuff selected according to arm circumference. cfPWV, as an index of AS, was assessed using a cuff-based volumetric displacement device (SphygmoCor XCEL, AtCor Medical, Sydney, NSW, Australia) [19]. The XCEL cuff was placed on the left upper arm to automatically measure brachial systolic blood pressure (SBP) and diastolic blood pressure (DBP) using standard oscillometric methods. The cuff was then immediately reinflated to a pressure below DBP. Next, an upper-thigh cuff was applied, and the XCEL system recorded volume-displacement waveforms at the femoral artery to cfPWV. All measurements were performed in the morning after ≥10 min of supine rest in a temperature-controlled room. Path length estimation followed the device protocol. In accordance with 2012 expert consensus guidelines, cfPWV > 10 m/s indicated increased AS [20]. Patients with cfPWV ≤ 10 m/s were categorized as the non-AS (control) group for subsequent analyses.

### Statistical analysis

Data analysis was carried out with SPSS version 25.0 (SPSS Inc., Chicago, IL, USA) and R version 4.2.2 (R Foundation for Statistical Computing, Vienna, Austria). The Kolmogorov–Smirnov test was used to assess the normality of the data. Normally distributed data were reported as means ± standard deviation and compared using the two-tailed independent Student’s t-test. Non-normally distributed data were summarized as medians with interquartile ranges and evaluated using the Mann–Whitney U test. Categorical variables were reported as counts and percentages and compared using the χ^2^ test. To identify variables independently associated with arterial stiffness, multivariable logistic regression was performed using covariates identified from univariate analyses. Model stability was evaluated using 1,000 bootstrap iterations. Variables with skewed distributions, including triglycerides, fasting glucose, HbA1c, albumin, BUN, creatinine, eGFR, UPCR, and phosphorus, underwent base-10 logarithmic transformation before regression. Associations between clinical variables and cfPWV, measured as a continuous variable, were evaluated using simple linear regression followed by multivariate forward stepwise linear regression. Variance inflation factor (VIF) analysis was used to assess multicollinearity; values below 10 indicate no concerning multicollinearity. Additional CKD stage-stratified multivariate models were evaluated to determine whether the associations were consistent across disease severity. Calibration was evaluated using calibration plots comparing predicted and observed risk, and clinical utility was assessed using decision curve analysis (DCA). Associations between serum ET-1 and clinical variables were analyzed using Spearman’s rank correlation coefficient. A two-tailed *p* value < 0.05 was considered statistically significant.

## Results

### Baseline characteristics

This study included 232 patients with non-dialysis CKD. Based on cfPWV measurements, 75 patients (32.3%) with cfPWV >10 m/s were classified as having AS, while the remaining 157 patients with cfPWV ≤10 m/s were categorized as the non-AS (control) group. The AS group had a higher prevalence of diabetes mellitus (*p* = 0.001) and hypertension (*p* = 0.003), older age (*p* = 0.044), and higher SBP and DBP (all *p* < 0.001), fasting glucose (*p* < 0.001), HbA1c (*p* = 0.008), UPCR (*p* = 0.009), and serum ET-1 levels (*p* < 0.001) than the control group. Sex distribution, prevalence of glomerulonephritis, use of anti-hypertensive drugs, anti-lipid drugs, and anti-diabetic drugs, and CKD stage did not differ significantly between groups (Table 1).

**Table 1.** Baseline demographic and clinical characteristics by aortic stiffness status.

Characteristics	All Patients (*n* = 232)	Control Group (*n* = 157)	Aortic Stiffness Group (*n* = 75)	*p* value
Age (years)	69.86 ± 10.89	68.87 ± 11.62	71.93 ± 8.87	0.044*
Height (cm)	160.21 ± 8.32	159.62 ± 8.07	161.44 ± 8.75	0.119
Body weight (kg)	67.47 ± 13.03	66.80 ± 13.02	68.87 ± 13.03	0.259
Body mass index (kg/m^2^)	26.22 ± 4.22	26.13 ± 4.11	26.39 ± 4.45	0.662
cfPWV (m/s)	9.37 ± 1.70	8.45 ± 1.00	11.31 ± 1.12	<0.001*
SBP (mmHg)	144.81 ± 20.93	140.55 ± 19.45	153.73 ± 21.22	<0.001*
DBP (mmHg)	77.33 ± 12.00	75.31 ± 11.86	81.56 ± 11.22	<0.001*
Hemoglobin (g/dL)	11.28 ± 1.98	11.15 ± 1.88	11.56 ± 2.17	0.138
Total cholesterol (mg/dL)	149.24 ± 41.16	147.33 ± 39.17	153.22 ± 45.07	0.310
Triglyceride (mg/dL)	121.5 (89.25–185.00)	118.00 (87.00–173.00)	132.00 (96.00–209.00)	0.102
LDL-C (mg/dL)	74.66 ± 28.08	73.91 ± 26.57	76.24 ± 31.14	0.556
Fasting glucose (mg/dL)	109.50 (95.00–138.00)	105.00 (92.50–129.00)	127.00 (102.00–155.00)	<0.001*
HbA1c (%)	6.20 (5.70–7.30)	6.00 (5.60–6.95)	6.60 (5.90–7.70)	0.008*
Albumin (mg/dL)	4.05 (3.80–4.20)	4.10 (3.80–4.20)	4.00 (3.80–4.20)	0.558
Blood urea nitrogen (mg/dL)	37.00 (28.00–51.00)	38.00 (28.50–51.00)	36.00 (28.00–51.00)	0.378
Creatinine (mg/dL)	2.40 (1.93–3.13)	2.40 (1.96–3.32)	2.40 (1.91–2.88)	0.375
eGFR (mL/min)	24.86 (18.61–32.69)	24.40 (18.32–31.75)	25.96 (19.32–35.63)	0.242
Spot UPCR (g/g)	0.74 (0.23–1.77)	0.72 (0.23–1.47)	0.83 (0.36–3.37)	0.009*
Total calcium (mg/dL)	9.27 ± 0.42	9.27 ± 0.42	9.28 ± 0.43	0.915
Phosphorus (mg/dL)	3.60 (3.30–4.10)	3.70 (3.30–4.10)	3.60 (3.30–4.10)	0.935
Endothelin-1 (pmoL/L)	0.42 ± 0.25	0.37 ± 0.21	0.54 ± 0.29	<0.001*
Female, *n* (%)	98 (42.2)	69 (43.9)	29 (38.7)	0.446
Diabetes mellitus, *n* (%)	100 (43.1)	56 (35.7)	44 (58.7)	0.001*
Hypertension, *n* (%)	135 (58.2)	81 (51.6)	54 (72.0)	0.003*
Glomerulonephritis, *n* (%)	53 (22.8)	39 (24.8)	14 (18.7)	0.295
Tubulointerstitial nephritis, *n* (%)	28 (12.1)	20 (12.7)	8 (10.7)	0.650
ARB use, *n* (%)	128 (55.2)	83 (52.9)	45 (60.0)	0.307
β-blocker use, *n* (%)	44 (19.0)	27 (17.2)	17 (22.7)	0.320
CCB use, *n* (%)	90 (38.8)	60 (38.2)	30 (40.0)	0.794
Statin use, *n* (%)	95 (40.9)	67 (42.7)	28 (37.3)	0.439
Fibrate use, *n* (%)	29 (12.5)	20 (12.7)	9 (12.0)	0.874
Metformin use, *n* (%)	83 (35.8)	53 (33.8)	30 (40.0)	0.354
Sulfonylurea use, *n* (%)	73 (31.5)	46 (29.3)	27 (36.0)	0.304
DDP-4 inhibitor use, *n* (%)	45 (19.4)	28 (17.8)	17 (22.7)	0.384
Insulin use, *n* (%)	26 (11.2)	16 (10.2)	10 (13.3)	0.478
SGLT2i use, *n* (%)	55 (23.7)	36 (22.9)	15 (25.3)	0.687
CKD stage 3	69 (29.7)	46 (29.3)	23 (30.7)	0.798
CKD stage 4	130 (56.0)	87 (55.4)	43 (57.3)	
CKD stage 5	33 (14.2)	24 (15.3)	9 (12.0)	

Normally distributed continuous variables are summarized as mean ± standard deviation, whereas skewed data are reported as median (interquartile range). Group differences were evaluated using the student’s t test or Mann–Whitney U test according to data distribution. Categorical variables are reported as numbers (percentages) and analyzed using the chi-square test. A *p*-value < 0.05 was considered statistically significant. Table abbreviations are defined as follows: cfPWV: carotid–femoral pulse wave velocity; SBP: systolic blood pressure; DBP: diastolic blood pressure; LDL-C: low-density lipoprotein cholesterol; HbA1c: glycated hemoglobin; eGFR: estimated glomerular filtration rate; UPCR: urine protein-to-creatinine ratio; ARB: angiotensin-receptor blocker; CCB: calcium-channel blocker; SGLT2i: sodium-glucose cotransporter 2 inhibitors; CKD: chronic kidney disease.

### Association between ET-1 and AS

Multivariable logistic regression showed that serum ET-1 was independently associated with AS. For each 0.01 pmoL/L increase in ET-1, the odds of AS increased (odds ratio [OR]: 1.022, 95% confidence interval [CI]: 1.008–1.037, *p* = 0.002). Age (OR = 1.042, 95% CI = 1.003–1.081, *p* = 0.033), DBP (OR = 1.054, 95% CI = 1.015–1.093, *p* = 0.006), and diabetes mellitus (OR = 3.269, 95% CI = 1.328–8.051, *p* = 0.010) were also independent predictors of AS (Table 2).

**Table 2.** Multivariable logistic regression model identifying independent predictors of aortic stiffness.

Variables	Odds ratio	95% confidence interval	*p* value
Endothelin-1, 0.01 pmoL/L	1.022	1.008–1.037	0.002*
Age, 1 year	1.042	1.003–1.081	0.033*
Diastolic blood pressure, 1 mmHg	1.054	1.015–1.093	0.006*
Diabetes mellitus, present	3.269	1.328–8.051	0.010*
Hypertension, present	0.694	0.249–1.932	0.484
Systolic blood pressure, 1 mmHg	1.014	0.988–1.042	0.294
Fasting glucose, 1 mg/dL	0.998	0.988–1.007	0.653
HbA1c, 1 %	0.918	0.647–1.304	0.633
Spot UPCR, 1 g/g	1.166	0.980–1.387	0.083

The model was adjusted for diabetes mellitus, hypertension, age, systolic and diastolic blood pressure, fasting glucose, glycated hemoglobin, UPCR, and serum endothelin-1. HbA1c: glycated hemoglobin; UPCR: urine protein-to-creatinine ratio.

* *p* < 0.05.

Bootstrap resampling with 1000 iterations confirmed the stability of ET-1, age, DBP, and diabetes mellitus as independent predictors of AS (Supplementary Table S1). Each 0.01 pmol/L increase in ET-1 was associated with a 2.2% rise in the odds of AS (coefficients (B) = 0.022). Age (*B* = 0.041), DBP (*B* = 0.052), and diabetes mellitus (*B* = 1.185) were significant predictors of AS. In contrast, hypertension, SBP, fasting glucose, HbA1c, and UPCR were not statistically significant after adjustment.

### Correlation between ET-1 and clinical variables

Serum ET-1 showed positive correlations with age (*r* = 0.235, *p* < 0.001), SBP (*r* = 0.294, *p* < 0.001), log-transformed fasting glucose (log-glucose, *r* = 0.171, *p* = 0.009), log-BUN (*r* = 0.144, *p* = 0.029), log-creatinine (*r* = 0.165, *p* = 0.012), log-phosphorus (*r* = 0.178, *p* = 0.007), and cfPWV (*r* = 0.358, *p* < 0.001). ET-1 was negatively correlated with hemoglobin (*r* = −0.267, *p* < 0.001), total cholesterol (*r* = −0.130, *p* = 0.048), log-albumin (*r* = −0.132, *p* = 0.045), and eGFR (*r* = −0.187, *p* = 0.004) (Table 3).

**Table 3.** Spearman correlation analysis between log-transformed serum endothelin-1 levels and clinical variables in patients with non-dialysis chronic kidney disease.

Variables	Spearman’s Correlation Coefficient	*p* value
Age (years)	0.235	<0.001*
Body mass index (kg/m^2^)	0.022	0.734
SBP (mmHg)	0.294	<0.001*
DBP (mmHg)	0.067	0.310
Hemoglobin (g/dL)	−0.267	<0.001*
Total cholesterol (mg/dL)	−0.130	0.048*
Log-Triglyceride (mg/dL)	0.043	0.519
LDL-C (mg/dL)	−0.091	0.168
Log-Glucose (mg/dL)	0.171	0.009*
Log-HbA1c (%)	0.057	0.387
Log-Albumin (mg/dL)	−0.132	0.045*
Log-BUN (mg/dL)	0.144	0.029*
Log-Creatinine (mg/dL)	0.165	0.012*
Log-eGFR (mL/min)	−0.187	0.004*
Log-UPCR (g/g)	0.066	0.317
Total calcium (mg/dL)	−0.063	0.343
Log-Phosphorus (mg/dL)	0.178	0.007*
Carotid-femoral PWV (m/s)	0.358	<0.001*

Values represent Spearman’s rank correlation coefficients (*r*). Positive and negative values indicate direct and inverse associations, respectively. Triglycerides, fasting glucose, HbA1c, albumin, blood urea nitrogen, creatinine, eGFR, UPCR, and phosphorus were log-transformed prior to analysis due to skewed distribution. Table abbreviations are defined as follows: SBP: systolic blood pressure; DBP: diastolic blood pressure; LDL-C: low-density lipoprotein cholesterol; HbA1c: glycated hemoglobin; BUN: blood urea nitrogen; eGFR: estimated glomerular filtration rate; UPCR: urine protein-to-creatinine ratio; PWV: pulse wave velocity. Statistical significance was set at *p* < 0.05.

### ET-1 is independently associated with cfPWV

Simple linear correlation analyses showed that cfPWV was associated with age, blood pressure, glycemic indices, UPCR, and ET-1. In the multivariable stepwise regression model, age (β = 0.279, adjusted R^2^ change = 0.040, *p* < 0.001), SBP (β = 0.226, adjusted R^2^ change = 0.252, *p* = 0.001), DBP (β = 0.291, adjusted R^2^ change = 0.029, *p* < 0.001), UPCR (β = 0.166, adjusted R^2^ change = 0.022, *p* = 0.003), and serum ET-1 (*β* = 0.196, adjusted R^2^ change = 0.045, *p* < 0.001) were independent predictors of cfPWV (Table 4). VIF values were <3.5 for all predictors, indicating no significant multicollinearity.

**Table 4.** Multivariable linear regression analysis of determinants of carotid–femoral pulse wave velocity in patients with non-dialysis chronic kidney disease.

Variables	Carotid-femoral pulse wave velocity (m/s)				
	Simple linear regression		Multivariable linear regression		
	*r*	*p* value	Beta	Adjusted R^2^ change	*p* value
Age (years)	0.238	<0.001*	0.279	0.040	<0.001*
Body mass index (kg/m^2^)	0.016	0.807	–	–	–
SBP (mmHg)	0.505	<0.001*	0.226	0.252	0.001*
DBP (mmHg)	0.386	<0.001*	0.291	0.029	<0.001*
Hemoglobin (g/dL)	0.064	0.330	–	–	–
Total cholesterol (mg/dL)	0.006	0.927	–	–	–
Log-Triglyceride (mg/dL)	0.116	0.079	–	–	–
LDL-C (mg/dL)	0.028	0.668	–	–	–
Log-Glucose (mg/dL)	0.220	0.001*	–	–	–
Log-HbA1c (%)	0.155	0.018*	–	–	–
Log-Albumin (mg/dL)	0.001	0.999	–	–	–
Log-BUN (mg/dL)	−0.123	0.060	–	–	–
Log-Creatinine (mg/dL)	−0.023	0.733	–	–	–
Log-eGFR (mL/min)	0.037	0.580	–	–	–
Log-UPCR (g/g)	0.231	<0.001*	0.166	0.022	0.003*
Total calcium (mg/dL)	−0.032	0.630	–	–	–
Log-Phosphorus (mg/dL)	−0.014	0.800	–	–	–
Endothelin-1 (pmoL/L)	0.358	<0.001*	0.196	0.045	<0.001*

Variables with skewed distributions (triglycerides, fasting glucose, HbA1c, albumin, blood urea nitrogen, creatinine, eGFR, UPCR, and phosphorus) were log-transformed prior to analysis. Table abbreviations are defined as follows: SBP: systolic blood pressure; DBP: diastolic blood pressure; LDL-C: low-density lipoprotein cholesterol; HbA1c: glycated hemoglobin; BUN: blood urea nitrogen; eGFR: estimated glomerular filtration rate; UPCR: urine protein-to-creatinine ratio. Statistical significance was set at *p* < 0.05.

### Stage-stratified analysis

When analyzed by CKD stage, serum ET-1 consistently remained an independent predictor of cfPWV in CKD stages 3, 4, and 5. This suggests that the association between ET-1 and vascular stiffness persists even as renal dysfunction progresses (Supplementary Table S2).

### Calibration and DCA

**Figure 1.**
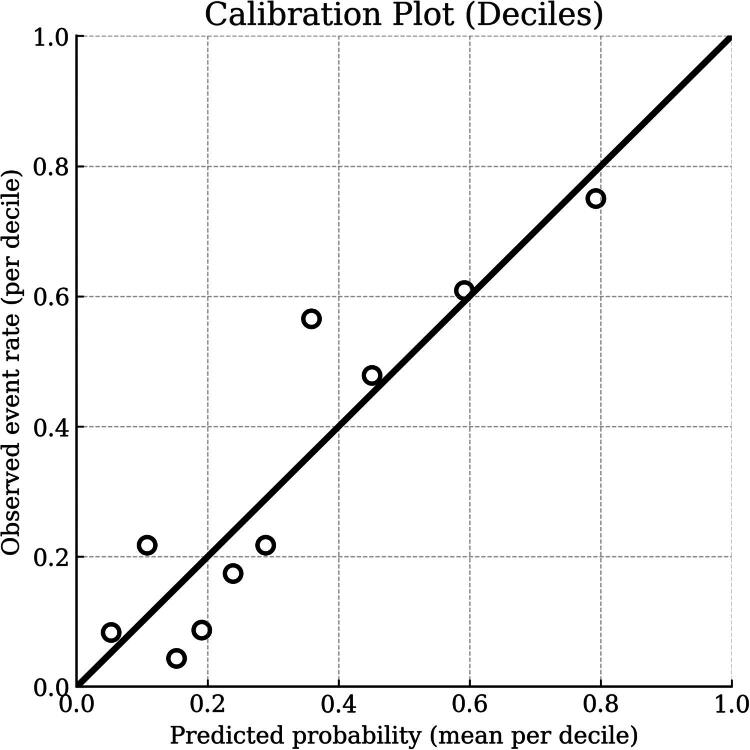
Calibration plot of the logistic regression model for predicting aortic stiffness, assessed using Hosmer–Lemeshow deciles. The plot compares observed proportions of aortic stiffness (y-axis) with model-predicted probabilities (x-axis) across 10 risk deciles. The 45° reference line represents perfect calibration, with points closer to the line indicating better agreement between predicted and observed risk.

**Figure 2.**
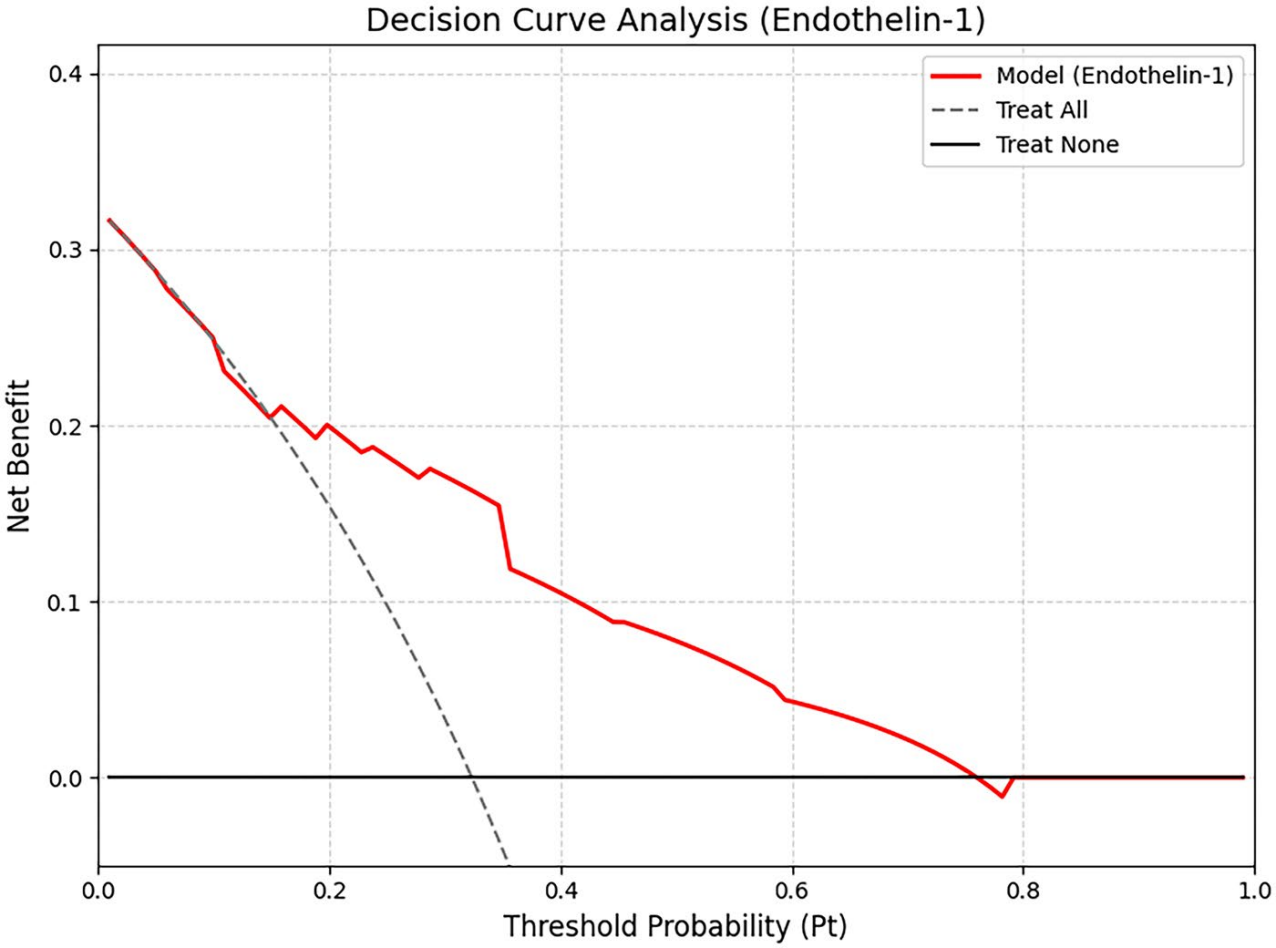
Decision curve analysis evaluating the clinical utility of serum endothelin-1 for identifying aortic stiffness in non-dialysis CKD patients. Net benefit is plotted against the threshold probability (pₜ). The red curve shows the model; the dashed gray line and solid black line represent the ‘treat-all’ and ‘treat-none’ strategies, respectively.

Calibration analysis showed strong agreement between predicted and observed risks (Figure 1), with the Hosmer–Lemeshow test indicating no lack of fit (χ^2^ = 12.893, *p* = 0.116). DCA confirmed that adding ET-1 to risk assessment provided a net clinical benefit across various threshold probabilities (Figure 2). The DCA analysis showed that the ET-1 model provided a higher net benefit than the default strategies across a wide range of threshold probabilities (approximately 0.1–0.8). This suggests that using ET-1 levels to risk-stratify patients confers significant clinical utility, enabling the identification of true-positive cases while minimizing unnecessary interventions (false positives) compared to a treat-all approach. The ET-1 prediction model demonstrated excellent calibration. The calibration intercept was 0.00 (95% CI: −0.31 to 0.31), indicating no systematic over- or under-estimation of risk. The calibration slope was 1.00 (95% CI: 0.69–1.30), reflecting perfect agreement between estimated probabilities and observed outcomes on average. The overall predictive performance was strong, with a Brier score of 0.166.

## Discussion

In this study of patients with non-dialysis CKD, elevated serum ET-1 levels were significantly associated with increased AS. ET-1 remained an independent predictor of AS after adjusting for age, blood pressure, diabetes mellitus, proteinuria, and renal function, with this association consistent across non-dialysis stages 3–5 of CKD. Moreover, ET-1 correlated independently with cfPWV, a continuous measure of AS, suggesting a dose-response association between ET-1 levels and vascular rigidity. Overall, these findings suggest that ET-1 reflects early vascular structural and functional alterations in CKD before the onset of end-stage renal disease.

Several established cardiometabolic factors were also associated with AS in this non-dialysis CKD cohort. Diabetes and hypertension were linked to greater vascular stiffness, consistent with the known impact of chronic metabolic and hemodynamic stress on the arterial wall [21]. Chronic hyperglycemia promotes the formation of advanced glycation end-products and oxidative injury, compromising the integrity of elastin and the function of vascular smooth muscle cells, thereby reducing arterial compliance [22]. Persistent hypertension contributes to medial remodeling and elastic fiber fragmentation, directly increasing AS [6,15]. Age was a significant determinant of cfPWV, reflecting cumulative vascular damage, including elastin fatigue, extracellular matrix remodeling, and early medial calcification [23]. Elevated SBP and DBP correlated with cfPWV, reinforcing the reciprocal relationship between arterial stiffness and hypertension [24]. Fasting glucose and HbA1c are also associated with vascular stiffening, underscoring the roles of both acute and chronic glucose exposure in vascular deterioration [25,26]. Moreover, the UPCR was positively associated with cfPWV, suggesting that proteinuria, a marker of glomerular and endothelial barrier impairment [27], may also reflect systemic vascular injury in CKD [28]. Together, these results confirm that traditional cardiovascular and renal risk factors remain closely tied to early vascular structural and functional alterations in CKD before dialysis dependence or overt cardiovascular events.

ET-1, the most potent endogenous vasoconstrictor, plays a critical role in regulating vascular tone and remodeling [29]. In this study, serum ET-1 independently predicted AS and cfPWV after adjusting for established risk factors, supporting its involvement in the pathogenesis of arterial stiffness in CKD. ET-1 primarily acts through endothelin A receptors (ETA receptors) on vascular smooth muscle cells, resulting in sustained vasoconstriction, inflammation, and oxidative stress [30]. Chronic activation of these pathways promotes extracellular matrix accumulation, collagen deposition, and elastin degradation, thereby accelerating arterial stiffening [31]. Previous studies have reported elevated ET-1 levels in vascular dysfunction conditions, such as hypertension, diabetes, heart failure, and atherosclerosis [32]. Positive correlations have been observed between ET-1 levels and pulse wave velocity or carotid intima–media thickness [33,34]. Mechanistically, ET-1 functions within a complex vasoactive network that is markedly dysregulated in CKD. Experimental and clinical studies have demonstrated that ET-1 reduces nitric oxide bioavailability, enhances oxidative stress, and amplifies angiotensin II-mediated vasoconstriction, thereby promoting endothelial dysfunction and vascular smooth muscle cell remodeling [35,36]. In CKD, these effects are further exacerbated by chronic inflammation, uremic toxin accumulation, and impaired renal clearance of ET-1. The combined actions of sustained vasoconstriction, extracellular matrix deposition, and elastin degradation contribute to the progressive stiffening of arteries. Although these interacting pathways were not directly assessed in the present study, the observed association between circulating ET-1 levels and cfPWV is biologically consistent with the established role of the endothelin system as a central mediator linking endothelial dysfunction to arterial stiffness in CKD, underscoring its potential role as a biomarker of early vascular dysfunction in non-dialysis CKD.

Clinically, these findings suggest that serum ET-1 may help identify patients with CKD who have a higher burden of AS. Current CKD guidelines emphasize cardiovascular risk stratification and the early identification of subclinical organ damage, but do not recommend specific circulating biomarkers for assessing vascular stiffness in non-dialysis CKD [37]. Accordingly, serum ET-1 should not be viewed as a routine screening or diagnostic test for AS. In this context, the diagnostic analyses demonstrated that ET-1 remained independently associated with AS after bootstrap validation, good calibration, and a modest net clinical benefit in DCA. Such performance is expected for a non-interventional biomarker and should be interpreted as exploratory rather than prescriptive. Within a multimodal cardiovascular risk assessment framework, ET-1 may help prioritize cfPWV evaluation or support earlier identification of subclinical vascular dysfunction in high-risk CKD populations, without replacing established vascular testing. From a pathophysiological perspective, the endothelin system plays a central role in endothelial dysfunction and vascular remodeling in CKD [38], providing biological plausibility for the observed association between circulating ET-1 levels and AS. Taken together, ET-1 may be considered an adjunctive, pathophysiology-reflecting biomarker within multimodal cardiovascular risk assessment frameworks in non-dialysis CKD, rather than a stand-alone screening or monitoring tool.

This study has several strengths. It is among the few to examine circulating ET-1 and AS in non-dialysis CKD, a population often overlooked for early vascular changes. The well-characterized cohort, standardized cfPWV measurement, and comprehensive biochemical and hemodynamic assessments strengthen internal validity. The use of multiple complementary statistical methods—including bootstrap validation, calibration analysis, and DCA—further supports the robustness and clinical relevance of the findings. However, several limitations should be acknowledged. First, the cross-sectional design precludes determination of whether ET-1 acts as a pathogenic mediator or a downstream marker of vascular injury; rather, our findings support its role as a clinically relevant indicator of vascular dysfunction in non-dialysis CKD. In addition, reliance on a single baseline measurement of serum ET-1 limits any inference regarding temporal relationships or causality, and the observed associations therefore reflect correlations at a single time point rather than disease progression. Second, the single-center setting and modest sample size may limit the generalizability of the findings. The absence of a non-CKD comparator group further restricts our ability to determine whether the observed association between ET-1 and AS is specific to CKD or represents a broader vascular phenomenon independent of renal dysfunction. Accordingly, our results should be interpreted within the context of a CKD population and may not be directly applicable to individuals without kidney disease. Finally, residual confounding related to pharmacological therapies cannot be excluded despite adjustment for major drug classes, and other vasoactive or inflammatory biomarkers were not measured, which may have provided additional mechanistic insights. Prospective cohort studies with repeated ET-1 measurements are needed to determine whether longitudinal changes in ET-1 precede, accompany, or follow the progression of aortic stiffness, and whether ET-1 dynamics are associated with subsequent cardiovascular events and mortality in CKD. Larger longitudinal and interventional studies are also required to validate the prognostic relevance of ET-1 and to explore whether modulation of the endothelin pathway may mitigate vascular stiffening in this population.

## Conclusions

In summary, this study demonstrates an independent association between circulating ET-1 levels and AS in patients with non-dialysis CKD. ET-1 should not be interpreted as a standalone diagnostic tool for vascular stiffness. Instead, these findings support ET-1 as an adjunctive, pathophysiology-reflecting biomarker that complements, rather than replaces, established cardiovascular risk factors and vascular assessments. While DCA suggests a net benefit in risk stratification, further prospective and longitudinal studies are required to confirm whether monitoring ET-1 provides incremental prognostic value in the clinical management of CKD.

## Supplementary Material

Supplementary Table.docx

## Data Availability

All data generated or analyzed during this study are fully included in this article. Any further inquiries may be directed to the corresponding author.
