# Supplementary material for: The correlation between serum endothelin-1 levels and aortic stiffness in patients with non-dialysis chronic kidney disease
Source: Ren Fail. 2026 Mar 12;48(1):2641961. doi: 10.1080/0886022X.2026.2641961 (PMC12983806; doi:10.1080/0886022X.2026.2641961)
Supplement: Supplementary Table.docx [file IRNF_A_2641961_SM5328.docx]

**Table S1.** Bootstrap validation of the multivariable logistic regression model identifying independent predictors of aortic stiffness.

| **Variables** | **B** | **Bca 95% CI** | ***p* Value** |
| --- | --- | --- | --- |
| Endothelin-1, 0.01 pmoL/L | 0.022 | 0.005, 0.042 | 0.002 |
| Age, 1 year | 0.041 | 0.005, 0.082 | 0.022 |
| Diastolic blood pressure, 1 mmHg | 0.052 | 0.015, 0.104 | 0.006 |
| Diabetes mellitus, present | 1.185 | 0.193, 2.325 | 0.010 |
| Hypertension, present | −0.365 | −1.620, 0.783 | 0.495 |
| Systolic blood pressure, 1 mmHg | 0.014 | −0.017, 0.048 | 0.353 |
| Fasting glucose, 1 mg/dL | −0.002 | −0.013, 0.010 | 0.641 |
| HbA1c, 1 % | −0.085 | −0.641, 0.380 | 0.664 |
| Spot UPCR, 1 g/g | 0.154 | −0.036, 0.397 | 0.077 |

A bootstrap resampling approach (1,000 repetitions) was applied to estimate bias-corrected and accelerated 95% confidence intervals. The reported coefficients (B) correspond to log-odds values from the fitted logistic regression model. Table abbreviations are defined as follows: BCa, bias-corrected and accelerated; CI, confidence interval; HbA1c, glycated hemoglobin; UPCR, urine protein-to-creatinine ratio.

**Table S2.** Stage-stratified multivariable linear regression analysis of determinants of carotid–femoral pulse wave velocity in patients with non-dialysis chronic kidney disease.

| **CKD stage** | **Predictor** | **B** | **SE** | **β** | **t** | **95% CI** | ***p* Value** |
| --- | --- | --- | --- | --- | --- | --- | --- |
| Stage 3 | SBP (mmHg) | 0.017 | 0.007 | 0.195 | 2.354 | 0.003, 0.030 | 0.020* |
|  | DBP (mmHg) | 0.032 | 0.011 | 0.235 | 2.913 | 0.010, 0.054 | 0.004* |
|  | ET-1 (pmoL/L) | 2.160 | 0.517 | 0.295 | 4.182 | 1.138, 3.183 | <0.001* |
|  | Log-UPCR (g/g) | 0.949 | 0.254 | 0.280 | 3.745 | 0.448, 1.451 | <0.001* |
|  | Age (years) | 0.042 | 0.012 | 0.262 | 3.535 | 0.018, 0.065 | 0.001* |
|  | GN (yes = 1) | −0.654 | 0.286 | −0.159 | −2.285 | −1.221, −0.087 | 0.024* |
| Stage 4 | SBP (mmHg) | 0.029 | 0.006 | 0.345 | 4.671 | 0.017, 0.042 | <0.001* |
|  | ET-1 (pmoL/L) | 2.070 | 0.540 | 0.283 | 3.833 | 1.001, 3.138 | <0.001* |
|  | Log-UPCR (g/g) | 0.870 | 0.244 | 0.257 | 3.560 | 0.386, 1.354 | 0.001* |
|  | Age (years) | 0.032 | 0.012 | 0.199 | 2.666 | 0.008, 0.055 | 0.009* |
| Stage 5 | ET-1 (pmoL/L) | 2.632 | 0.663 | 0.491 | 3.969 | 1.274, 3.991 | <0.001* |
|  | Age (years) | 0.077 | 0.018 | 0.510 | 4.194 | 0.040, 0.115 | <0.001* |
|  | GN (yes = 1) | 0.981 | 0.425 | 0.287 | 2.306 | 0.110, 1.853 | 0.029* |
|  | Calcium (mg/dL) | −0.781 | 0.343 | −0.275 | −2.280 | −1.483, −0.079 | 0.030* |

Analyses were performed separately for CKD stages 3, 4, and 5, treating cfPWV as a continuous dependent variable. Variables with skewed distributions were log-transformed prior to regression, and predictors were selected using forward stepwise modeling within each CKD stage. Covariates were prespecified based on clinical relevance and included diabetes, hypertension, GN, tubulointerstitial nephritis, age, body mass index, SBP, DBP, hemoglobin, total cholesterol, log-triglycerides, LDL-cholesterol, log-glucose, log-HbA1c, log-albumin, log-BUN, log-creatinine, log-eGFR, log-UPCR, calcium, and log-phosphorus. Table abbreviations are defined as follows: cfPWV, carotid–femoral pulse wave velocity; SBP, systolic blood pressure; DBP, diastolic blood pressure; ET-1, endothelin-1; HbA1c, glycated hemoglobin; BUN, blood urea nitrogen; eGFR, estimated glomerular filtration rate; UPCR, urine protein-to-creatinine ratio; GN, glomerulonephritis. Statistical results include regression coefficients, standard errors, standardized coefficients, t-values, two-sided *p*-values, and 95% confidence intervals. Statistical significance was set at *p* < 0.05.
